# Whole-Genome Sequencing Detection of Ongoing *Listeria* Contamination at a Restaurant, Rhode Island, USA, 2014

**DOI:** 10.3201/eid2208.151917

**Published:** 2016-08

**Authors:** Jonathan S. Barkley, Michael Gosciminski, Adam Miller

**Affiliations:** Rhode Island Department of Health, Providence, Rhode Island, USA

**Keywords:** listeria, listeriosis, whole-genome sequencing, WGS, environmental contamination, contamination source, epidemiologic investigation, environmental investigation, bacteria, bacterial pathogen, food processing, food service establishments, outbreak, pulsed-field gel electrophoresis, PFGE, foodborne infections

## Abstract

In November 2014, the Rhode Island Department of Health investigated a cluster of 3 listeriosis cases. Using whole-genome sequencing to support epidemiologic, laboratory, and environmental investigations, the department identified 1 restaurant as the likely source of the outbreak and also linked the establishment to a listeriosis case that occurred in 2013.

Infection with *Listeria monocytogenes*, a foodborne bacterial pathogen, causes listeriosis, which can lead to severe illness, typically among persons with compromised immune systems and pregnant women and their fetuses. The pathogen can survive at high salt concentrations and grow at refrigeration temperatures (*1*). These properties enable the bacteria to persist in food processing and food service establishments for extended periods. Listeriosis has a long incubation period (3–70 days), making exposure recall difficult. Retail delicatessens are a potential source of *L. monocytogenes* because they hold ready-to-eat foods at refrigeration temperatures; however, a risk assessment by the United States Department of Agriculture’s Food Safety and Inspection Service suggests that thorough sanitization of food contact surfaces, proper maintenance of equipment and facilities, safe product handling practices, and good employee practices to avoid cross-contamination can help prevent listeriosis cases associated with retail food establishments (*2*).

Since 1998, PulseNet (http://www.cdc.gov/pulsenet/index.html) has used pulsed-field gel electrophoresis (PFGE) to look at genetic differences in *L. monocytogenes* subtypes and to identify outbreaks. However, distantly related strains can appear indistinguishable by PFGE; thus, greater differentiation may be needed to distinguish between outbreak and sporadic cases of listeriosis. Whole-genome sequencing (WGS) offers an opportunity to further discriminate between strains and identify outbreaks. WGS has historically been used retrospectively to provide additional insight into outbreak investigations (*3*). However, since September 2013, WGS has been performed on all clinical *L. monocytogenes* isolates identified in the United States by the Centers for Disease Control and Prevention (Atlanta, GA) and several state public health laboratories (*4*). *L. monocytogenes* is a good candidate for WGS because it causes a relatively rare condition that can result in serious illness, it has a small genome that is relatively easy to analyze, and epidemiologic surveillance and food regulatory program components for the bacterium are strong (*5*).

Data obtained from WGS has been analyzed using whole-genome multilocus sequence typing (wgMLST), a technique that examines allelic differences from thousands of loci, and ≈96% of *L. monocytogenes* coding sequences have been identified as loci in the wgMLST scheme (S. Stroika, Centers for Disease Control and Prevention, pers. comm., 2016 Jan 29). To discriminate between strains and identify outbreaks, alleles within the coding sequence (i.e., loci) are compared with ≈178 reference genomes. A unique combination of alleles at each locus specifies the sequence type, which enables comparison of isolates (*6*); the smaller the number of allelic differences between isolates, the more related they are.

The Rhode Island Department of Health (RIDOH) attempts interviews and, when applicable, conducts environmental investigations for all reports of listeriosis. Each year during 2011–2013, RIDOH received ≈3 reports of listeriosis, most of which were sporadic cases. However, in November 2014, a cluster of cases was detected from laboratory reports and examined using WGS in conjunction with epidemiologic, laboratory, and environmental investigations. Isolates were confirmed to be *L. monocytogenes* and submitted for PFGE analysis. The Centers for Disease Control and Prevention performed WGS on clinical isolates; the Food and Drug Administration performed WGS on food isolates.

## The Investigation

During October 27–November 5, 2014, RIDOH’s Center for Acute Infectious Disease Epidemiology was notified of 3 *L. monocytogenes*–infected persons residing in the same city. The 3 case-patients were all non-Hispanic white persons >60 years of age; 2 had an immunocompromising condition. Interviews conducted by the Center for Acute Infectious Disease Epidemiology identified a single common restaurant visited by the 3 patients. RIDOH’s Center for Food Protection performed inspections and collected food and environmental samples at the establishment.

PFGE analysis showed that clinical *L. monocytogenes* isolates from the 3 case-patients shared an identical, common PFGE pattern (Figure). To determine the relationship between the isolates, RIDOH collaborated with federal partners to conduct WGS. Results of wgMLST showed that the isolates were closely related (0–5 allelic differences) (Figure) and a close genetic match (median allelic differences 4) to a clinical isolate from a 2013 patient, who was reinterviewed and reported eating at the same restaurant. A sliced prosciutto sample from the restaurant tested positive for *L. monocytogenes*, and PFGE patterns for this isolate matched those for isolates from the 2013 and 2014 case-patients. Results of wgMLST showed that the isolate from the prosciutto differed by 0–5 alleles (median 3) from the 2014 clinical samples and by 0–11 alleles (median 4) from the 2013 clinical sample (Figure). Sequences for the isolates were uploaded to GenBank (*7*) (clinical isolates: accession nos. SAMN02400177, SAMN03253348–49, SAMN03253359; isolate from prosciutto: accession no. SAMN03218571).

**Figure:**
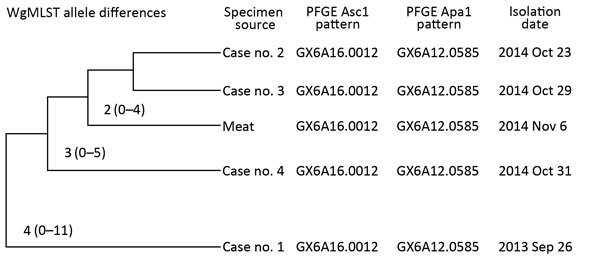
Median (minimum–maximum) allele differences and pulsed-field gel electrophoresis (PFGE ) patterns for *Listeria monocytogenes* isolates from clinical and food samples associated with a 2014 cluster of listeriosis cases and a 2013 listeriosis case, Rhode Island, USA. Allele differences were determined by whole-genome multilocus sequence typing (wgMLST). Adapted from data provided by the Enteric Diseases Laboratory Branch, National Center for Emerging and Zoonotic Infectious Diseases, Centers for Disease Control and Prevention (Atlanta, GA, USA).

A total of 10 food and environmental food samples were initially collected from the restaurant. Swab samples were obtained from the food slicer, preparation tables, and walk-in cooler. Environmental investigation of the restaurant identified issues related to control of *L. monocytogenes*: the temperature of the refrigerated unit that held sliced meat and other food items was elevated (52°F [11°C]), and cleanliness issues were observed with the preparation tables and slicer. An additional 19 environmental samples were later collected from the establishment; however, the refrigerated unit and preparation tables had been replaced, so additional swab samples could not be collected from those surfaces. The sample of sliced prosciutto was the only *L. monocytogenes*–positive sample identified at the restaurant; however, just 1 of the 2014 case-patients reported eating prosciutto (in an antipasto salad) at the restaurant. Other foods reported included green salad and coleslaw.

RIDOH tested a sample of prosciutto from an unopened package from the establishment and collaborated with the Food Safety and Inspection Service to see if the processing plant had recently tested positive for *L. monocytogenes*. The sample tested negative, and no positive tests had been reported at the plant in at least 1 year.

## Conclusions

Epidemiologic, environmental, and laboratory investigation results implicated a restaurant with sanitation issues and improper sliced meat storage as the likely source of a multiyear listeriosis outbreak. A long incubation period makes WGS an effective technology to use during listeriosis outbreak investigations and to identify outbreak-associated cases originally believed to be sporadic cases. This technology can help overcome difficulties associated with investigating listeriosis cases and can be useful for the investigation of other pathogens. In this investigation, WGS (wgMLST) helped link the 2013 listeriosis case, which was originally believed to be a sporadic case, to the 2014 outbreak. Furthermore, given that the 4 isolates had a common PFGE pattern, this technology increased confidence that the restaurant, which was the only common restaurant among the 4 patients, was the source of the outbreak. The allelic differences observed are consistent with slow, spontaneous mutation occurring over a long period due to persistent contamination.

There is no set number of allelic differences used to determine whether clusters of cases are part of actual outbreaks (*8*). Thus, WGS is not sufficient by itself to identify outbreaks and must be performed in conjunction with epidemiologic, laboratory, and environmental investigations (*8*,*9*). In the investigation we describe, WGS was used in this supporting role. The close relationship that WGS showed between the clinical isolates and the isolate from meat provides additional evidence that the restaurant was the likely source of contamination for the cases in 2013 and 2014.

Our findings support the need to control *L. monocytogenes* at retail food establishments. Storing meat at <41°F (5°C) can prevent ≈9% of listeriosis cases (*2*). In addition, retail delicatessens and food establishments can prevent *L. monocytogenes*–associated illnesses among customers by controlling cross-contamination, cleaning and sanitizing food contact surfaces, and eliminating environmental niches.
